# P120 regulates beta-catenin nuclear translocation through E-cadherin endocytosis in ventilator-induced lung injury

**DOI:** 10.18632/oncotarget.13724

**Published:** 2016-11-30

**Authors:** Changping Gu, Chenyang Dai, Yongtao Sun, Mengjie Liu, Yuelan Wang, Xinyi Wu

**Affiliations:** ^1^ Department of Anesthesiology, Qianfoshan Hospital of Shandong University, Jinan, Shandong, People's Republic of China; ^2^ Department of Ophthalmology, Qilu Hospital of Shandong University, Jinan, Shandong, People's Republic of China

**Keywords:** p120, β-catenin, nuclear translocation, E-cadherin endocytosis, VILI, Pathology Section

## Abstract

Mechanical stretch induces epithelial barrier dysfunction by altering the location and degradation of cellular junction proteins. p120-catenin (p120) is a cell-cell junction protein known to protect against ventilator-induced lung injury (VILI) that results from improper ventilation of patients. In this study, we sought to determine the role of p120 in VILI and its relationship with the cellular response to mechanical stretch. Mouse lung epithelial cells (MLE-12) transfected with p120 siRNA, p120 cDNA, or E-cadherin siRNA were subjected to 20% cyclic stretch for 2 or 4 hours. Wild-type male C57BL/6 mice were transfected with p120 siRNA-liposome complex to delete p120 *in vivo* and then subjected to mechanical ventilation. Cyclic stretch induced p120 degradation and the endocytosis of E-cadherin, which induced β-catenin translocation into the nucleus, a key event in lung injury progress and repair. These findings reveal that by reducing β-catenin nuclear translocation through inhibition of E-cadherin endocytosis, p120 protects against ventilator-induced lung injury.

## INTRODUCTION

Mechanical ventilation is an effective therapy for acute lung injury (ALI) and acute respiratory distress syndrome (ARDS) patients, but inappropriate ventilation can cause lung injury [1, 2]. Mechanical ventilator-induced lung injury (VILI) has been linked to the destruction of the epithelial monolayer and the production of proinflammatory cytokines, both of which eventually induce pulmonary edema and systemic inflammatory response [3-6].

Cyclic stretch is the most studied form of mechanical stretch. It induces structural and cytosolic changes in alveolar epithelial cells, which in turn lead to alveolar epithelial barrier dysfunction and pulmonary hyperpermeability [7-10]. The integrity of the epithelial monolayer is dependent on the association of junction proteins, which include E-cadherin, β-catenin, α-catenin, and p120. Previous studies have shown that p120 played an important regulatory role in inhibiting inflammation [11] and epithelial gap formation [10] induced by mechanical ventilation. These studies suggest that p120 binds directly to the cytoplasmic domain of E-cadherin to protect the integrity of the cell junction [10, 12]. Moreover, Villar and colleagues showed that activation of Wnt/β-catenin signaling induced the accumulation of β-catenin in the cell nucleus, which plays a central role in lung injury progress and repair [13].

In this study we sought to determine the effect of p120 on the epithelial monolayer damage caused by mechanical ventilation. We explored the effects of overexpression and knockdown of p120 on gap formation and localization of other gap proteins by subjecting cultured cells and mice to cyclic stretch and mechanical ventilation, respectively.

## RESULTS

### Cyclic stretch alters the expression, distribution, and association of junction proteins

**Figure 1:**
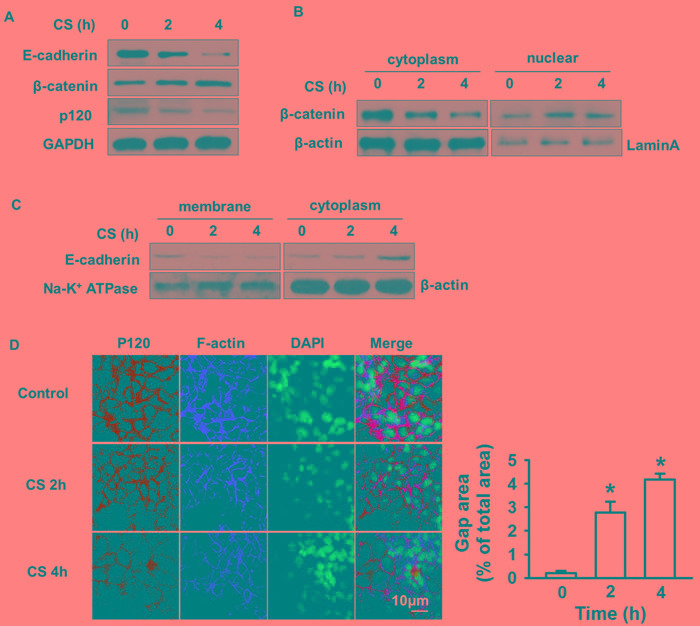
Cyclic stretch induced degradation and redistribution of junction proteins MLE-12 cell monolayers on Flex cell plates were subjected to 20% cyclic stretch for 2 or 4 h. **A.** Western blot showed p120, E-cadherin decreased while β-catenin increased after cyclic stretch. **B.** The expression of β-catenin in cytoplasm and nucleus was determined by Western blot. **C.** Membrane and cytoplasmic protein were subjected to Western blot to determine E-cadherin endocytosis. **D.** Immunofluorescence staining and quantification data revealed the MLE-12 cells gap formation. MLE-12 cells were fixed and stained with anti-p120 followed by Alexa-conjugated secondary antibody (green) and actin cytoskeletal remodeling was stained with Texas red-conjugated phalloidin (red). The nucleus (blue) was stained with DAPI. Scale bar = 10 μm. *p < 0.05 *versus* control group. Experiments were repeated at least three times.

After 20% cyclic stretch, the expression of E-cadherin and p120 decreased in a time dependent manner, while total β-catenin remained unchanged. After 4 h cyclic stretch, the protein levels of E-cadherin and p120 had decreased a lot (Figure 1A). We separated and extracted cytoplasmic and nuclear protein. We found that β-catenin decreased in the cytoplasm and increased in the nucleus after cyclic stretch (Figure 1B). The level of E-cadherin was reduced in the membrane but increased in cytoplasm after 20% cyclic stretch (Figure 1C). p120 and F-actin immunofluorescence showed cyclic stretch increased epithelial gap formation (Figure 1D). Quantitative analysis of stretch-induced gap formation suggested that the total gap area after 4 h cyclic stretch significantly increased compared with the control group, which indicated that cyclic stretch destroyed the integrity of the epithelial monolayer consistent with previous results [10].

### p120 prevents epithelial gap formation by inhibiting β-catenin nuclear translocation

**Figure 2:**
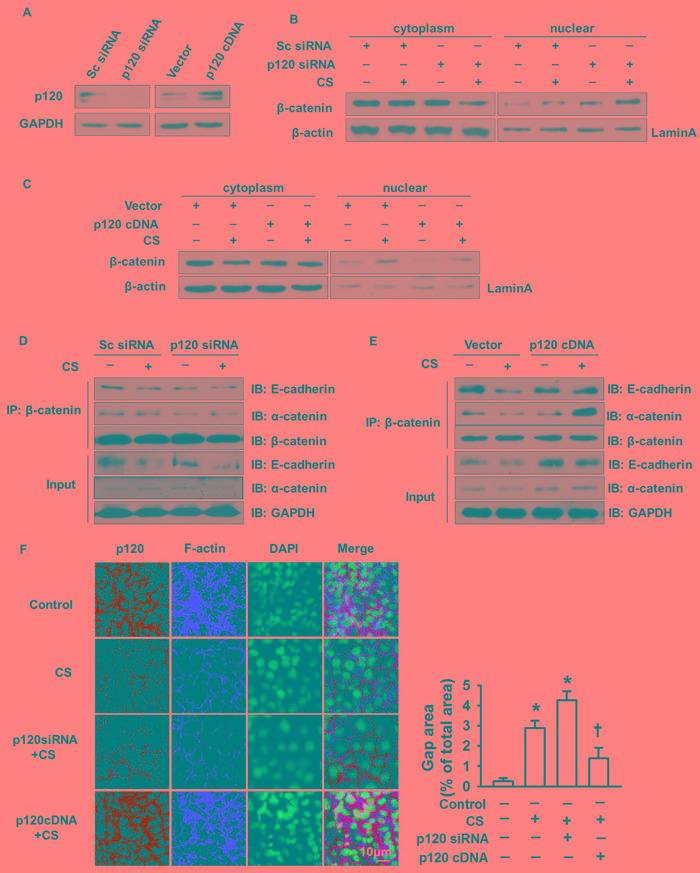
The regulatory role of p120 in β-catenin nuclear translocation induced gap formation MLE-12 cells were transfected with p120 siRNA or p120 cDNA. Forty eight hours post-transfection, cells were exposed to 20% cyclic stretch for 2 h. **A.** Successful transfection was detected by Western blot. **B.**, **C.** The nuclear translocation of β-catenin in p120 siRNA and p120 cDNA group after 20% cyclic stretches. **D.**, **E.** The association of β-catenin with α-catenin and E-cadherin were examined by immunoprecipitation after 2 h cyclic stretch in p120 siRNA or p120 cDNA transfection groups. **F.** Immunofluorescence and quantification data showed the gap formation in p120 siRNA group and p120 cDNA group. Scale bars = 10 μm. *p < 0.05 *versus* control group, +*p* < 0.05 *versus* CS group. Experiments were repeated at least three times.

To explore the role of p120 in β-catenin nuclear translocation-induced gap formation, we knocked down and overexpressed p120 using p120 siRNA and cDNA overexpression constructs, respectively. At 48 h post-transfection, cells were lysed and successful transfection was determined by western blot. The expression of p120 was decreased 90% in p120 siRNA group and increased 70% in p120 cDNA group compared with control group (Figure 2A). Loss of p120 induced β-catenin nuclear accumulation, which was enhanced by 2 h cyclic stretch (Figure 2B). Overexpression of p120 reversed the nuclear accumulation of β-catenin (Figure 2C). Immunoprecipitation demonstrated that p120 depletion under cyclic stretch caused the disassociation of β-catenin from E-cadherin and α-catenin while their interactions were restored in the p120 overexpression group (Figure 2D-2E). Immunofluorescence and quantitative analysis confirmed that epithelial gap formation was enhanced in the p120 siRNA knockdown group while reduced in p120 overexpression group under cyclic stretch (Figure 2F).

### p120 inhibits β-catenin nuclear translocation through E-cadherin endocytosis after cyclic stretch

**Figure 3:**
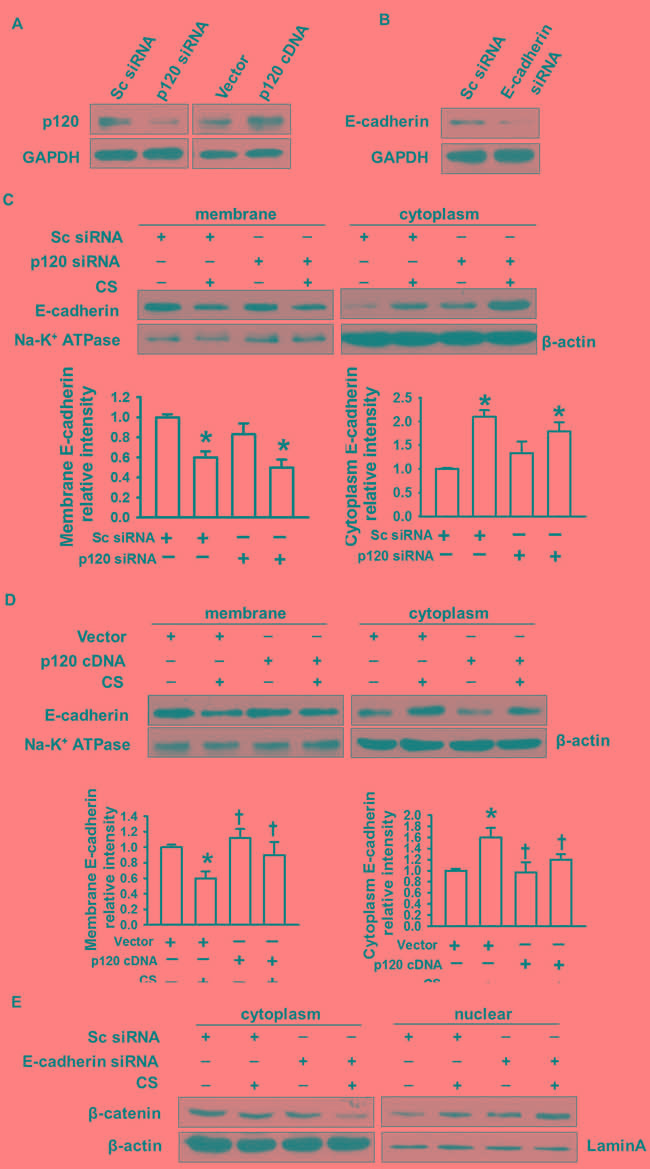
The regulatory role of p120 in E-cadherin endocytosis-induced β-catenin nuclear translocation MLE-12 cells were transfected with p120 siRNA, E-cadherin siRNA or p120 cDNA. 48h post-transfection, cells were exposed to 20% cyclic stretch for 2 h. **A.** Successful p120 siRNA and p120 cDNA transfection were detected by Western blot. **B.** Successful E-cadherin siRNA transfection was detected by Western blot. **C.**, **D.** E-cadherin endocytosis in p120 siRNA and p120cDNA group under 20% cyclic stretches. **E.** The effects of E-cadherin depletion on β-catenin nuclear translocation. *p < 0.05 *versus* control group, +*p* < 0.05 *versus* CS group. Experiments were repeated at least three times.

To explore the mechanism of p120 in regulating the nuclear translocation of β-catenin, we determined the distribution of E-cadherin in the membrane and cytoplasm in p120 knockdown or overexpression groups under 20% cyclic stretch. Successful transfection was shown in Figure 3A. We found that loss of p120 caused E-cadherin endocytosis and that E-cadherin expression was decreased in the membrane while increased in the cytoplasm after 20% cyclic stretch for 2 h (Figure 3C). Additionally, we observed that overexpression of p120 reversed E-cadherin endocytosis (Figure 3D).

To further investigate the relationship between β-catenin nuclear translocation and E-cadherin endocytosis, we transfected MLE-12 cells with E-cadherin siRNA. Successful transfection was shown in Figure 3B. β-catenin nuclear translocation was enhanced in the E-cadherin siRNA group under 20% cyclic stretch for 2 h (Figure 3E). Therefore, as in the E-cadherin siRNA experiment, p120 degradation reduced the level of membrane E-cadherin and then promoted the nuclear translocation of β-catenin.

### Loss of p120 *in vivo* causes pulmonary inflammation

**Figure 4:**
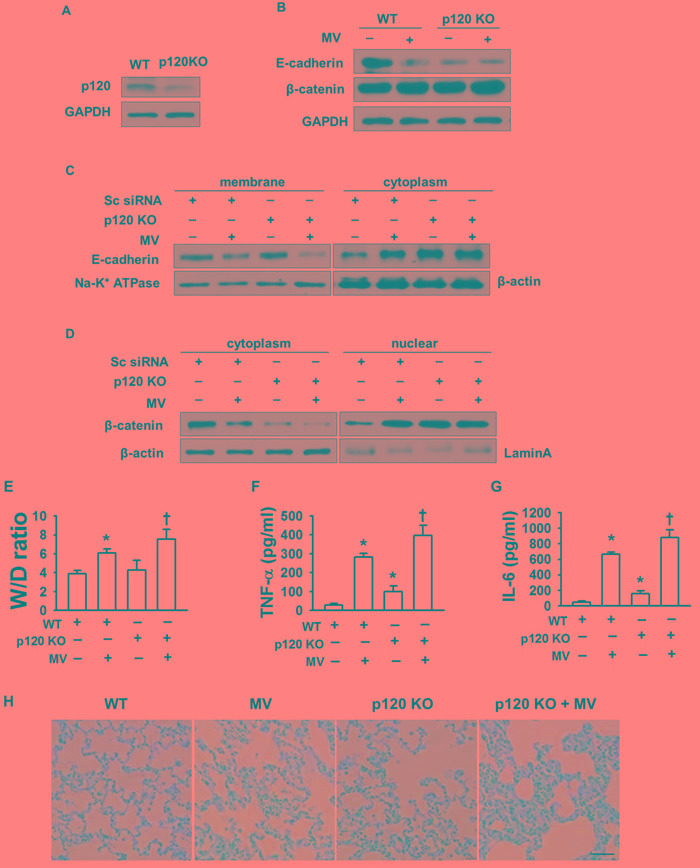
The role of p120 in preventing pulmonary edema in VILI *in vivo* Mice were transfected with p120 siRNA-liposome complex before exposure to mechanical ventilation for 2 h. A normal tidal volume of 7 ml/kg with a frequency of 120 breath /min was administered to the control group. A tidal volume of 28 ml/kg with a frequency of 60 breath /min was administered to the high tidal volume group (MV). **A.** Successful transfection of p120 siRNA was confirmed by Western blot of lung homogenates in mice. **B.** Effects of p120 depletion on the level of junction proteins. **C.** Effects of p120 depletion on E-cadherin endocytosis **D.** Effects of p120 depletion on the nuclear translocation of β-catenin *in vivo*. **E.** Lung W/D ratio. **F.**, **G.** cytokines production in BAL fluid. **H.** The pathological changes in p120 depleted (knock out) mice with or without high tidal volume ventilation. Scale bar = 50 μm. *p < 0.05 *versus* control group, +*p* < 0.05 *versus* MV group. Experiments were repeated at least three times.

We next investigated the mechanism of p120 regulation of cytoskeletal proteins *in vivo*. We prepared p120 siRNA-liposome complexes and administered them to mice through the retinal vein plexus. After mechanical ventilation with varying tidal volumes for 2 h, lung tissue and bronchoalveolar lavage (BAL) fluid were obtained. Western blot results showed that p120 *in vivo* was depleted by about 80% (Figure 4A). The level of E-cadherin was also reduced after mechanical ventilation (Figure 4B). Moreover, we found that p120 knock out (KO) caused E-cadherin endocytosis. E-cadherin decreased in the membrane and increased in the cytoplasm after mechanical ventilation (Figure 4C). Consistent with the results from our cell culture experiments, the level of β-catenin was reduced in the cytoplasm and increased in the nucleus in p120 KO group with or without mechanical ventilation (Figure 4D).

Next, we determined the effects of p120 and mechanical ventilation on W/D lung weight ratio in order to quantify pulmonary edema. Our W/D ratio results revealed that the wet lung weight increased in the high tidal volume ventilation group and the ratio in high tidal volume group was similar to previously reported ratios (17, 32). After depletion of p120, the W/D ratio was enhanced significantly compared with the control group in the high tidal volume group (Figure 4E). p120 depletion caused pulmonary edema in the high tidal volume group.

Moreover, the production of the cytokines TNF-α and IL-6 in BAL fluid was significantly increased in the high tidal volume ventilation group and was further increased in the p120 depletion group after high tidal volume mechanical ventilation (Figure 4F-4G).

Consistent with the cytokine results, hematoxylin and eosin (HE) staining showed characteristic inflammatory damage in the high tidal volume ventilation group: edema, leukocyte infiltration, and changed lung tissue structure. All of these types of damage were increased in the p120 depletion group after high tidal volume ventilation, which indicated an essential role of p120 in protection from ventilator-induced lung injury (Figure 4H).

## DISCUSSION

VILI is caused primarily by mechanical ventilator-induced pulmonary edema and the lung inflammatory response. p120, a prototypic member of a growing subfamily of Armadillo-domain proteins [14], has been revealed to contribute to the integrity of cell-cell junction and to the inflammatory response [10, 11, 14]. In this study, we illustrate a new role for p120 in regulating the integrity of the lung epithelial monolayer.

We have shown that cyclic stretch induces p120 degradation, which in turn promotes E-cadherin endocytosis and β-catenin nuclear translocation. Our results are consistent with the findings of a previous study which also showed that the association of these junction proteins is destroyed after mechanical stretch [10]. Additionally, E-cadherin endocytosis (caused by p120 degradation) was shown to contribute to the nuclear translocation of β-catenin which is enhanced in mice transfected with E-cadherin siRNA after cyclic stretch. Moreover, depletion of p120 *in vivo* increased lung inflammation after high tidal volume ventilation, which was consistent with previous studies [7, 10]. In vivo, the Wet/Dry (W/D) ratio and inflammatory cytokine levels (including tumor necrosis factor α (TNF-α) and interleukin-6 (IL-6)) were significantly increased in the high tidal volume ventilation group in p120 depleted mice. Lung tissue pathologic changes including pulmonary alveolar edema, leukocyte infiltration, and damaged lung tissue were observed in p120 depleted mice. All of these results suggested that p120 played an essential role by reducing β-catenin nuclear translocation which contributed to the integrity of epithelial barrier function.

p120 protects the integrity of cell junctions by maintaining association with E-cadherin [14-18]. Previous studies have suggested that cyclic stretch could activate c-Src kinase which in turn leads to the degradation of p120 and destroys adherent junctions. Moreover, p120 could inhibit RhoA activation and prevent tight junctions from being destroyed during cyclic stretch. However, the mechanism of p120 in protecting other adherent junction proteins had not been elucidated.

β-catenin is a ubiquitously expressed protein that is located at cell-cell adherent junctions in association with α-catenin and other cadherins [19]. These interactions bind these proteins to the cytoskeleton [20]. While in the adherent junction, cadherin recruits β-catenin molecules into the intracellular regions. We observed that E-cadherin endocytosis decreased the ability of cadherin to recruit β-catenin. This caused accumulation of β-catenin in the nucleus and prevented it from associating with α-catenin which directly bonded to the actin filaments. The β-catenin - α-catenin complex could thus physically bridge cadherin with the actin cytoskeleton.

β-catenin participates in the Wnt signaling pathway and is involved in regulating cell proliferation, differentiation, survival, and fate [21]. The mechanism of p120 degradation which induces β-catenin nuclear translocation might be related with these mechanisms. We found that p120 degradation enhanced E-cadherin endocytosis and nuclear translocation of β-catenin. Thus, the association of β-catenin with α-catenin and E-cadherin was destroyed in the absence of p120 degradation, consistent with epithelial gap formation determined by immunofluorescence in this study.

This study reveals a new mechanism of p120 in regulating cell-cell junctions in VILI. Mechanical stretch induces p120 degradation, which causes E-cadherin endocytosis. Without the recruiting effect of E-cadherin, β-catenin translocates from the cytoplasm to the nucleus. Thus, β-catenin disassociates from α-catenin and E-cadherin, all of which contribute to the epithelial gap formation and lung inflammation. These results suggest potential new therapeutic targets for the prevention and treatment of VILI.

## MATERIALS AND METHODS

### Constructs and antibodies

p120 siRNA (pool)and E-cadherin siRNA (pool) were purchased from Dharmacon. p120 cDNA was obtained from the University of Illinois at Chicago. All primary antibodies and normal IgG are purchased from Santa Cruz Biotechnology. 4′, 6-diamidino-2-phenyl indole dihydrochloride (DAPI), Alexa-labeled secondary antibodies and Alexa 546 phalloidin were purchased from Invitrogen.

### Animals

All animal studies were approved by the Animal Ethics Committee of Shandong University. Forty wild-type male C57BL/6 mice (25-30 g) were obtained from the Animal Care and Use Committee of Shandong University. Mice were housed in specific pathogen-free conditions, and used in experiments at 8-12 weeks of age.

### Cell culture and cyclic stretch

Mouse lung epithelial cells (MLE-12) were obtained from the American Type Culture Collection (Manassas, VA). The cells were plated at a density of 8 × 10^5 ^cells/well on collagen I-coated flexible bottom BioFlex plates in DMEM with 10% FBS and grown to confluence in the incubator. After 48 h of culture, cells were transferred to serum-free for 2 h before stretch. Experimental plates with cell monolayers were subjected to cyclic stretch using FX-4000T Flexcell Tension Plus system (Flexcell International, McKeesport, PA). Cyclic stretch was conducted at 20% change in basal membrane surface area which corresponds to 80% of total lung capacity [22-24] with a frequency of 30 cycles/min (0.5 Hz) and a stretch-to-relaxation relation of 1:1 [10]. Non stretched cells (static cells) were used as controls.

### p120 or E-cadherin knock down and overexpression in MLE-12 cells

p120 siRNA, a pool of three target-specific 20-25nt siRNA and scrambled (sc) siRNA, p120 1A cDNA, or the empty vector were transfected into 70 % confluent MLE-12 cells according to the manufacturer's protocol. Lipofectamine 2000 was used as the transfection regent. The same protocol was used in E-cadherin siRNA transfection experiments. All experiments were conducted after 48 h transfection and successful transfection was verified by Western blot analysis.

### Liposome preparation and *in vivo* gene delivery

We used liposome based vectors verified to deliver p120 siRNA *in vivo* through retinal vein plexus injection [25]. We mixed dimethyl dioctadecyl ammonium bromide and cholesterol (1:1 molar ratio) in a round-bottom flask, and evaporated the solvent using a Rotavapor evaporator (Brinkmann, Westbury, NY), rotating the flask until the mixture was dry. The final deposit was dissolved in 5% glucose. Each animal received 150 μl of the siRNA-liposome complexes containing 50 μl p120 siRNA (50nM) and 100 μl liposomes through retinal vein plexus injection. Successful transfection of p120 siRNA was confirmed by Western blot of lung homogenates two days after transfection.

### Experimental protocols

In order to deplete p120, C57BL/6 mice were treated with freshly prepared siRNA-liposome complexes through retinal vein plexus injection. Two days later, mice were anesthetized with intraperitoneal injection of 75mg/kg of a ketamine/xylazine mixture. Then, mice were tracheotomized and treated with mechanical ventilation. Mice that received a normal tidal volume of 7 ml/kg with a frequency of 120 breath /min were regarded as the control group. A tidal volume of 28 ml/kg with a frequency of 60 breath /min was administered to high tidal volume group [26-28]. After 4h of mechanical ventilation, some mice were injected with 1ml cold PBS three times through the trachea cannula. BAL fluid was collected and used for cytokine detection. Some mice were sacrificed and their lungs were taken to determine the Wet/Dry weight ratio and the p120 depletion efficiency *in vivo* by Western blot analysis.

### Western blot

Western blot analysis of the cell lysate and the lung tissue was performed as previously described [10, 27, 28]. MLE-12 cells and lung tissues were lysed on ice in RIPA buffer with the addition of 1mM protease inhibitor, 1 mM sodium orthovanadate, and 1 mM PMSF for 20min. Then, the cell lysate and tissue homogenates were sonicated. After centrifugation, protein concentration in supernatants was detected using bicinchoninic acid protein assay kit (Pierce, Rockford, IL). Equal amounts of protein from different groups were loaded onto 10% SDS-PAGE. Then, the proteins were transferred from the gel to polyvinylidene fluoride membranes. The membrane strips were blocked in 5% non-fat milk (TBS, 0.1% Tween-20, 5% non-fat milk, PH 8.8) for 1 h at room temperature. Then, primary antibodies were added and incubated overnight at 4 °C. After washing with TBST, the membrane strips were treated with relevant secondary antibody for 1 h at room temperature. Finally, Proteins on the membrane strips were detected with ECL SuperSignal reagent (Pierce). Relative band densities of the various proteins were analyzed using National Institutes of Health ImageJ Software.

### Immunoprecipitation

Immunoprecipitation analysis was performed using A/G PLUS-agarose beads (Santa Cruz Biotech) according to the manufacture's protocol. MLE-12 cells and lung tissues were lysed with prepared RIPA buffer. The supernatants of the cells and tissue homogenates were precleared by adding 1mg normal IgG together with protein A/G PLUS-agarose beads for 30 min, followed by incubation overnight with anti-β-catenin antibodies together with 20 μl protein A/G PLUS-agarose beads on a shaker at 4 °C. The final immunoprecipitates were washed and dissolved in 20 μl SDS-PAGE sample buffer for immunoblot analysis.

### Immunofluorescence staining

At the end of stretch, MLE-12 cells were washed and fixed with 4% paraformaldehyde. Then, the cells were permeabilized for 10 min with 0.2% Triton X-100 in HBSS. After blocking with 5% BSA in PBS, the cells were incubated with anti-p120 antibody overnight at 4 °C with following incubation with relative Alexa-labeled secondary antibody for 30 min at room temperature. For negative control, cells were incubated with normal IgG. Actin filaments were stained with Texas red-conjugated phalloidin (1:40 dilution in PBS). Cell nuclei were labeled with DAPI (2 μg/ml) for 20 min and then the cells were rinsed for three times. Flex membranes were excised with a razor blade and mounted on glass slides with ProLong Antifade Mountain Medium (Molecular Probes). The co-localization of junction proteins and the cell gap were recorded with a laser scanning confocal microscope (Zeiss LSM 510 META). Images were differentially segmented between gaps and cells based on image grayscale levels. Quantitative analysis of gap formation was performed using Image J software.

### Cytokine assay

Levels of TNF-α and IL-6 in BAL fluid were detected by using commercial ELISA kits (R&D System and BD Biosciences) according to the manufacturer's instructions [27]. Each value represents the mean of triplicate determinations.

### Wet/Dry weight ratio

The W/D lung weight ratio was determined to quantify pulmonary edema. One lobe of right lung tissue was obtained after mechanical ventilation. Blood and water were removed from the lung surface. The lung tissues were weighed and then dried in an oven to a constant weight (60°C for 72 h). Finally, we measured the dry weight and calculated the wet/dry ratio.

### Histological analysis

The lung tissues from mice subjected to different tidal volumes were obtained after successful transfection with p120 siRNA. Tissues were fixed in 10% neutral-buffered formalin and then subsequently embedded in paraffin. Finally, 5 mm thick sections were stained with HE using a standard protocol. Pathological changes were observed under light microscopy.

### Statistical analysis

One-way analysis of variance (ANOVA) with a post-hoc Turkey's pair-wise comparison was used for statistical analysis among different groups. Student's *t*-test was performed for paired samples. Parameter changes between different groups over time were evaluated by a two-way ANOVA with repeated measurements. All results are expressed as mean ± SEM, and a *p* < 0.05 was considered statistically significant.
